# The experiences and acceptability of a novel multimodal programme for the management of fibromyalgia: A qualitative service evaluation

**DOI:** 10.1002/msc.1672

**Published:** 2022-07-15

**Authors:** Suzanne McIlroy, Bethany Vaughan, Heather Crowe, Lindsay Bearne

**Affiliations:** ^1^ Physiotherapy Department King's College Hospital NHS Foundation Trust London UK; ^2^ Health Psychology Section Psychology Department King's College London London UK; ^3^ School of Population Health and Environmental Sciences King's College London London UK; ^4^ Population Health Research Institute St George's University of London London UK

**Keywords:** acceptability, exercise, fibromyalgia, physiotherapy, qualitative, self‐management

## Abstract

**Introduction:**

Fibromyalgia guidelines recommend multi‐modal, non‐pharmacological interventions but there is limited evidence on the optimal programme. The Fibromyalgia Active Management and Exercise programme (FAME) aimed to improve function and quality of life. It consisted of 12 sessions delivered by a multidisciplinary team and incorporated education, exercise, Cognitive Behavioural Therapy and mindfulness approaches.

This qualitative service evaluation explored the experience and acceptability of FAME from the perspective of the patients' and healthcare practitioners' (HCP).

**Methods:**

All patients and HCP involved in the first FAME programme were invited to attend either one audio‐recorded focus group or an individual semi‐structured interview. Topic guides were developed a priori. Data were transcribed verbatim and analysed thematically.

**Results:**

Thirteen participants (six HCP (three physiotherapists, two nurses, one psychologist)) and seven patients (mean age 46 (7.5) years, all female,) were enroled. FAME was acceptable to HCP participants but not to all patient participants. Where patient participants understood and anticipated the aims of FAME, the programme was found to be acceptable. Whereas, patient participants who did not fully understand the aims of the programme reported lower acceptability. Three themes were generated: *expectations and preparation for FAME, the value of social* *support*, and *FAME as a learning opportunity*. The themes could be explained by five constructs of the Theoretical Framework of Acceptability.

**Conclusion:**

FAME was acceptable to HCPs but not to all patient participants. Patient and HCP participants valued social support and regarded this as central to their learning. Further adaptation of FAME is required to optimise acceptability.

## 1 INTRODUCTION

Fibromyalgia is a chronic disorder characterised by widespread persistent pain with a broad spectrum of symptoms including fatigue, irritable bowel syndrome, difficulty concentrating and low mood (Arnold et al., 2019). These symptoms reduce quality of life by impacting physical and psychological functioning, personal relationships and ability to undertake activities of daily living (Bennett et al., 2007; Skaer, 2014) resulting in high personal, healthcare and societal burden (Boonen, 2004; Soni et al., 2020).

Fibromyalgia affects approximately 3%–7% of the general population (Collin et al., 2017; Jones et al., 2015). More women than men are affected (female:male ratio 3:1) (Queiroz, 2013). The pathophysiology of fibromyalgia is poorly understood. It is proposed that symptoms are caused by disordered pain regulation with associated neuroendocrine changes in the central and peripheral nervous system. Physical and emotional trauma, particularly in childhood or adolescence, stressful life events and psychosocial conditions (e.g. depression, lower socioeconomic status and education levels) may be predisposing factors (Ghavidel‐Parsa & Bidari, 2021).

Management of fibromyalgia aims to reduce symptoms and increase function (Macfarlane et al., 2016). Multi‐modal, non‐pharmacological interventions are recommended (Macfarlane et al., 2016; Pearson et al., 2021; UK, 2021) due to the diverse symptoms of fibromyalgia and limitations of single treatment modalities (Bernardy et al., 2010; Bidonde et al., 2019; Wang et al., 2018). A recent systematic review including 13 studies suggests that multidisciplinary interventions that incorporate education, exercise and psychological approaches to support self‐management may be effective (Llàdser et al., 2021). Yet there is paucity of evidence and lack of consensus on optimal content, format and mode of delivery of interventions (Bidonde et al., 2019; Geraghty et al., 2021). Additionally, physiotherapists and other healthcare workers report frustration and helplessness when managing people with fibromyalgia (Briones‐Vozmediano et al., 2013). This leads to role ambiguity, lack of confidence and reluctance to treat people with fibromyalgia (Roitenberg & Shoshana, 2021). New models of care are required (Doebl et al., 2021).

In 2019 The Fibromyalgia Active Management and Exercise programme (FAME) was developed at one NHS foundation Trust in London. FAME was a 12‐week group outpatient programme (12 people/group) that aimed to improve function and quality of life by facilitating self‐management and physical activity in people with fibromyalgia. It was designed and refined by a multi‐disciplinary clinical team. The format and duration was adapted from a successfully running 12‐week programme for people with persistent low back pain with the content adapted following a literature review (Busch et al., 2013; Karjalainen et al., 1999; Lee et al., 2014; Macfarlane et al., 2016). It consisted of weekly two‐hour interactive sessions delivered by multidisciplinary healthcare professionals. Each session focussed on a different topic such as exercise, stress management (e.g. mindfulness), and was underpinned by behaviour change approaches such as Cognitive Behavioural Therapy (supplement file 1). Eligible patients (supplement file 2) were enroled onto FAME by their treating clinician (e.g. physiotherapists, rheumatologists, and pain team clinicians).

For a programme to be successfully implemented it must be acceptable to both the participants and the healthcare practitioners (HCP) delivering the programme. Acceptability is defined as a “Multi‐faceted construct that reflects the extent to which people delivering or receiving a health care intervention consider it to be appropriate, based on anticipated experiential cognitive or emotional responses to the intervention” (Sekhon et al., 2017). Previous studies have measured acceptability from a quantitative perspective for example, drop‐out rates (Bernardy et al., 2018; Häuser et al., 2011) and, whilst important, does not provide a deep understanding of the important issues and possible solutions. The Theoretical Framework of Acceptability (TFA), considers acceptability to consist of seven constructs (Affective Attitude; Burden; Ethicality; Intervention Coherence; Opportunity Costs; Perceived Effectiveness; Self‐efficacy) (Sekhon et al., 2017) that may be assessed quantitatively and qualitatively to gain a comprehensive measure of acceptability. Robustly evaluating acceptability of FAME, from the perspective of people who receive and deliver it is an essential initial component before the scalability and sustainability is considered (Klaic et al., 2022).

The study aimed to explore patients and healthcare professionals' experiences of the FAME programme, drawing on the theoretical framework of acceptability and identify areas of programme refinement.

## 2 METHODS

The study is reported in accordance with the Consolidated criterion for reporting qualitative research checklist (COREQ) (Tong et al., 2007).

## 3 ETHICAL APPROVAL

Ethical approval was not required but the protocol registered by King's College Hospital service evaluation and audit committee.

### 3.1 Study design

This service evaluation included qualitative interviews and focus groups with attendees and healthcare professionals (HCP) delivering the FAME programme at one NHS foundation Trust in the United Kingdom.

## 4 PROCEDURE

### 4.1 Participants and recruitment

All attendees at the first FAME group cohort and HCPs that contributed to the delivery of the programme were invited to participate in this qualitative service evaluation. They were provided with written information about the purpose of the evaluation and invited to attend either one of two focus groups (1 patient, 1 HCP group) at the NHS Hospital Foundation Trust.

If patients or HCPs were unable to attend a focus group, they were offered an individual semi‐structured telephone interview (face‐to‐face or via telephone dependent upon participant preference) with one researcher.

Interviews and focus groups followed a topic guide developed a *priori*, by the authors, and informed by previous literature (Bearne et al., 2017; Galea Holmes et al., 2017) and the Theoretical Framework of Acceptability (Sekhon et al., 2017) (supplementary file 3). The topic guides were reviewed and refined in consultation with a Psychologist and piloted in a focus group of student healthcare professionals. No changes to the guide were made. This data was not included in the final analysis.

Participants were asked open‐ended questions about their expectations, experiences, and suggestions for refinements for the FAME programme. Core questions were included in all focus groups and the opportunity to explore relevant but unanticipated areas and reflections were provided. The focus group facilitator or interviewer used probes to encourage participants to express their views. The focus group facilitator ensured all participants had the opportunity to add their views to the discussion by inviting comments from each attendee, if needed. Field notes were made during each focus group by an observer and after each focus group and interview by the facilitator/interviewer to provide contextual information.

### 4.2 Research team and reflexivity

The research team comprised a female Consultant Physiotherapist involved in the development of FAME (SMc), and two female student MSc Pre‐registration physiotherapy students (BV, HC). None of the researchers were involved with the clinical management of any interviewee or the delivery of the FAME programme.

### 4.3 Data generation

Participants completed a sociodemographic questionnaire that included age, gender, ethnicity (self‐reported and categorised based on UK census categories (Office for National Statistics, 2021), duration of symptoms (patient participants only) and/or profession and duration of experience working with patients with persistent pain (HCP participants only)). All focus groups were conducted by one of two lead facilitators (SMc or HC) and attended by two other researchers, one acted as a co‐facilitator and the other as an observer who made field notes. All interviews were conducted by one of two researchers (BV, HC). The participants were aware of the research teams backgrounds.

All recordings were audio‐recorded, transcribed verbatim by an external transcription agency (patient focus group) or by the researchers, anonymised, and checked for accuracy against the original recordings by the researchers. Data generation ceased when all participants willing to participate had been included.

## 5 DATA ANALYSIS

An inductive thematic analysis approach was conducted (Braun & Clarke, 2006). This six‐stage approach comprised of (i) familiarisation with text; (ii) coding within the computer package NVIVO 11 (*QSR International Ltd., Southport, UK*); (iii) categorisation of codes and (iv) building of themes and subthemes; (v) defining and naming of themes; (vi) identification of extracts and quotes to illustrate themes.

BV and HC were responsible for the primary coding. The initial themes were developed in consultation with SMc and reviewed with the research team to discuss how they related to the aims and theoretical framework of acceptability (SMc, LB). A summary of the main findings were emailed to a sub‐sample of the participants (*n* = 3) to review, check for resonance and add to the interpretation of the findings.

### 5.1 Findings

Thirteen patients and seven HCPS were invited to take part in this study between March and July 2019. Seven patient participants (all female, age range between 32 and 56 years) and six HCP participants (four female) attended one of two 60‐min focus groups (one patient; one HCP) or a 30‐min semi‐structured interview (two telephone interviews with patients, one face‐to‐face interview with an HCP). Table 1 contains the participant demographics.

**TABLE 1:** Participant sociodemographic and clinical characteristics

**Patient participants**					
**Participant code**	**Age (Years)**	**Ethnicity**	**Duration of Symptoms (Years)**	**Number of sessions attended**	
P1	52	Other mixed	9	9	
P2	45	White British	Unknown	12	
P3	47	Black British ‐Caribbean	Unknown	8	
P4	48	Other: Ecuadorian	9	10	
P5	44	White British	18	10	
P6	56	Black British ‐ Caribbean	14	7	
P7	32	Black British ‐ African	8	6	

**Health Care Professional participants**					
**Participant Code**	**Sex**	**Ethnicity**	**Profession**	**Experience working in the profession (Years)**	**Experience working with patients with persistent pain (Years)**
**HCP1**	Male	White British	Nurse	30	25
**HCP2**	Male	White British	Psychologist	10	10
**HCP3**	Female	White British	Physiotherapist	5.5	3.5
**HCP4**	Female	White British	Physiotherapist	9.5	9.5
**HCP5**	Female	White British	Physiotherapist	6	8
**HCP6**	Female	White British	Nurse	20	17

Abbreviation: HCP, Health care professional.

### 5.2 Themes

Overall patient participants reported mixed views about the FAME programme. Most patient participants appreciated some aspects of FAME, particularly the opportunity to meet other people with fibromyalgia but revealed that, at times, the FAME programme did not make sense to them or align with their expectations of treatment. This affected their experience of the programme, willingness to engage with the programme content and satisfaction with the programme.

All HCP participants reported that FAME was acceptable to them, although they perceived that further discussion with patient participants prior to referral to the programme, alongside minor changes to the programme, could optimise its acceptability and effectiveness for some patient participants.

**FIGURE 1:**
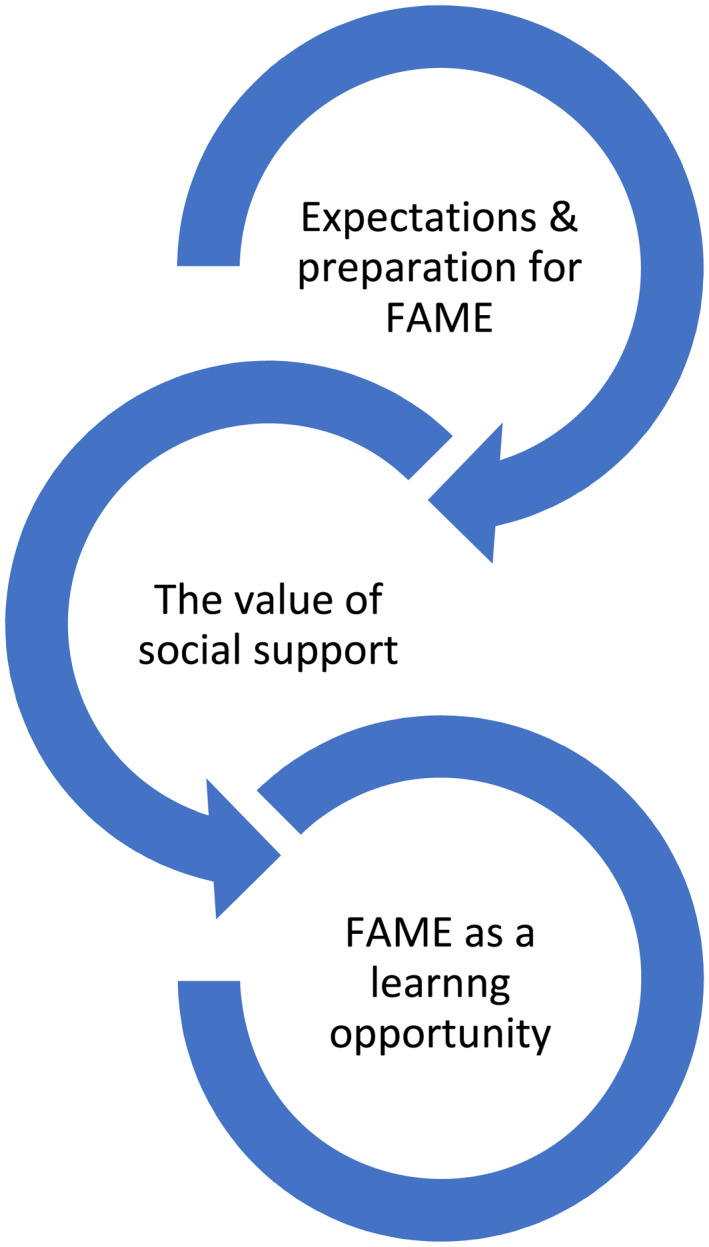
The inter‐related themes of: *expectations and preparation for FAME, the value of social support,* and *FAME as a learning opportunity*

Three themes were identified (*expectations* and *preparation for FAME, the value of social* *support*, and *FAME as a learning opportunity,* Figure 1).

### 5.3 Theme 1 expectations and preparation for FAME

Patient participants' expectations of FAME varied and were influenced by their perception of the aims and content of the programme. Some patient participants had little idea of what to expect of the programme or their expectations did not align with their experiences of the programme. These participants explained that FAME did not make sense to them and did not meet their needs or answer their questions about their condition. Some patient participants suggested that their referral to programme was not a shared treatment decision, and they did not regard FAME as an acceptable treatment for them.

*I generally was sent here because we was hoping I’d get some answers to some things that was going on with my health but unfortunately I didn’t get that* ‐P3

In contrast, some patient participants anticipated the format and content of the programme and welcomed the opportunity to share their experiences with other patient participants. In these patient participants FAME met their needs and expectations by offering information, support, and complementary management techniques

*I got what I expected and that was to be in a community around people that understood me and what I was going through and we could share our pain and suffering and not feel like we were misunderstood*‐ P1

*I just really wanted to see, for me, what is it that they can offer in regards to relief. Not necessarily just tablets. What I could get out of it in regards to exercise groups and you know like therapy groups* –P6

The need to align FAME attendees understanding of their condition, their needs and treatment expectations with the FAME programme content was recognised by all patient participants. However, patient participants had mixed views whether people with newly diagnosed fibromyalgia would benefit more than those with longstanding fibromyalgia. Some patient participants recommended that the programme was suitable for people regardless of the duration of their condition fibromyalgia whereas one patient participant recommended that those newly diagnosed would benefit more.

*You know, you’ve got to work out… what your target group is. You can’t [have] newbies and oldbies in the same group and expect them to achieve the same thing* ‐P5

*So, if someone’s newly diagnosed, new to all of this, … [it] will be of benefit and will educate and help those people. People that have had it ten/twenty years plus have already covered everything* ‐P5

*I don’t actually think that like* ‐P3

HCP participants unanimously expressed attendees at FAME should be informed about the programme aims and content prior to starting the programme so that they were prepared to engage with new perspectives and treatment options. They considered that this discussion and shared treatment decision making was appropriate regardless of duration since diagnosis. HCP participants perceived that if FAME attendees were not willing to engage with the new perspectives offered by the programme or ready to change their management approach, then FAME may not be suitable for, or acceptable to them. HCP participants were concerned that attendees who were not willing to engage with the programme content influenced the group interactions and potentially undermined the delivery of the programme and how it was received by others.

*they need to … be open to trying that more kind of psychological approach and treatment and kind of learn strategies for managing things, otherwise you just get those constant barriers in the way which is just not helpful in a group setting because then it interferes with other people* – HCP3

All HCP participants reflected on the importance of preparing potential attendees for FAME to ensure they were able to benefit from FAME. HCPs suggested that additional pre‐programme psychological assessment may be helpful to identify those potential attendees that were most likely to respond to the programme however, they reported that they may not have the resources to do this. It was also proposed that written information about FAME or a taster session followed by an active ‘opt‐in’ requirement may help potential attendees to understand the aims, format and content, and to ‘buy‐in’ to FAME. HCP and patient participants agreed that lack of written information hindered the coherence of FAME and patient participants felt that they didn't know what to expect and so were not able to fully engage or prepare for sessions.

*you kind of need to get them sort of geared up to what the class is going to offer, because I find if you, you kind of look down a list and see they’ve got fibromyalgia, … and you say oh we’ve got this class do you want to go to it? Without giving them any background or sort of prepping them for it, then they aren’t ready to take on the change or accept change.* –HCP5

### 5.4 Theme 2: The value of social support

FAME offered participants the opportunity to receive support from other people with lived experience of their condition. Both patient and HCP participants recognised the value of peer support. However, joining a group of people with the same condition was a new experience for most patient participants, and they valued the opportunity to share their experiences of fibromyalgia. Patient participants reported that they felt understood by peers and gained a sense of belonging. Patient participants reported that their experience of peer support affected their attitude towards FAME. This influenced their attendance at FAME sessions as they valued the emotional support from their peers. These established friendships continued after the FAME programme finished as part of an ongoing independent peer support network.

*I think I more come because I’d made friends and it can be very lonely having fibro* – P5

However, some patient participants reported that their peer support network was threatened by inconsistent attendance of some attendees and that this affected their sense of a shared community. Whilst social support from the FAME group was highly valued by patient participants, some patient participants identified that the programme did not completely remove the need for individual treatments to help them manage specific symptoms.

HCP's also valued peer support from the multi‐professional group of FAME facilitators. This increased their confidence to deliver the FAME programme and manage challenging situations that developed.

*I find just having the support from the MDT [multidisciplinary team] … it was quite nice to have a bit of a debrief, either via email with everyone or after the class*. –HCP5

Both HCP and patient participants felt that some attendees negatively influenced the group sessions and overpowered other group members. HCP participants found this difficult to moderate and address the perceived ‘*unrelentingly’* challenges about the session content. Some patient participants reported that this conflict marred their experience of FAME and reduced their engagement with, and the acceptability of, the programme. One patient participant considered the HCPs to be overwhelmed by the FAME attendees who challenged the FAME content and management approach. Attendee selection, expectation setting and agreeing clear group rules were proposed by the HCP participants to support positive group dynamics and learning.

*there were some weeks when I felt overpowered by particular people moaning and that wasn’t productive*‐ P6

Inviting family members to attend FAME was viewed positively by patient participants. They reported that this allowed family members to gain an insight into what it is like to live with fibromyalgia and learn how they can provide support at home. Patient participants perceived that this increased the effectiveness of FAME. Conversely, some HCP participants considered family members attending the FAME sessions burdensome because they asked questions about themselves and their own health conditions. Some HCP participants reported that this was distracting and added complexity to facilitating the group discussions. To address this issue, one HCP identified that guidance on family member attendance and reiteration of the group's rules at each session may resolve this issue.

Patient participants views on the support provided by the HCPs varied. Some patient participants reported HCPs worked with/alongside them to understand and address any problems they had and that this increased their self‐efficacy to self‐manage.

*I was thinking that I was, I would be able to sort my problem because he was beside me*– P4

In other cases, patient participants felt HCPs did not understand their individual needs and revealed that a more individualised approach would aid the effectiveness and the acceptability of the programme. Both participant groups identified that different staff taking each session within one FAME programme negatively affected the therapeutic relationship. HCPs acknowledged that consistent staffing was important for continuity and effectiveness of the programme. Patient participants reported that changing staff meant that some HCPs did not get to know their individual needs and sometimes completed sessions in a perfunctory manner.

*sometimes it would be somebody completely different who may or may not have prior knowledge of the group as well and I think that’s the difficulty in delivering this kind of programme. I think one of the key things is that there has to be a consistent staff group* ‐HCP6

### 5.5 Theme 3: FAME as a learning opportunity

FAME was recognised as a learning opportunity for most participants. Patient participants learnt about their condition, the spectrum of symptoms and new ways of managing these.

HCPs participants reported that working within a new multidisciplinary team meant that they developed their understanding of Fibromyalgia, new ways of communicating concepts to attendees and about wider services available for people with fibromyalgia. For HCPs participants, working with this patient group provided other areas of learning, for example, managing challenging group dynamics and being able to address preconceptions about living with the condition.

Both groups of participants found the core components of FAME coherent (education, exercise, and mindfulness) but the value they attributed to each component varied. They recommended including an expert patient in future iterations of the programme to further enhance the opportunity for learning and the effectiveness of FAME.

*I think there’s that old thing isn’t it, walk a mile in my shoes and unless you live it… as we’ve said we’re learning it from each other as well, so where are you going to get that information from? It’s from the people that live it.*‐ P3

Some of the patient participants appreciated learning from the HCPs and valued their professional advice and ability to tailor the content to their individual needs. However, other patient participants reported that some staff were not able to tailor information in the group setting. This affected their attitude towards and perceived effectiveness of FAME.

*thinking a little bit more about what we need as human beings, as a person in different needs*‐ P4

Some patient participants found the breadth topics and the general nature of the content did not meet their needs and wanted more individual support. Conversely, some patient participants recognised that not all topics were relevant to them but were willing to engage with FAME until they could identify topics that were useful. For example, the experience of and enthusiasm for exercise varied between patient participants. Some patient participants reported that they were pushed to complete exercise, and they did not understand why exercise was such a key component of FAME. These patient participants considered that exercise was too burdensome and reported that it exacerbated their symptoms. For example, one patient participant stated that

*I did do it [exercise] and it nearly killed me after that. I couldn’t walk from there to there.*‐P3

In contrast, other patient participants found exercise was a positive experience and found attending FAME made exercise accessible to them. They reported that they would like more exercise within FAME. One patient participant reported that FAME taught everyone to exercise

*in the best way that suits you and [that you] were comfortable whilst doing the exercises. So that was really helpful* – P7

Universally, patient participants found that learning about, and practising, mindfulness was useful to help manage the pain and stress associated with fibromyalgia and valued the time spent on this during the programme. HCP participants agreed that mindfulness training was a valuable part of the programme as it provided patients a self‐management technique and was an activity that everyone was interested and participated in. One participant suggested that mindfulness provided

*a way of getting a sense of control also, controlling your mind… and not letting the pain take over … it kind of does help you know ‐* P7

## 6 DISCUSSION

This is one of the first qualitative service evaluations to explore the experiences and the acceptability of FAME from the perspective of both the patients with fibromyalgia and HCP. The HCP participants broadly viewed FAME positively whereas the patients' opinions were more diverse and influenced by their expectations of FAME.

The perceptions and acceptability of the programme were explained by three themes: *expectations and preparation for FAME, the value of social* *support*, and *FAME as a learning opportunity*.

The Theoretical Framework of Acceptability (TFA) (Sekhon et al., 2017) can be used to understand the acceptability of the FAME programme from both patient and HCP participant perspectives. The framework comprises seven constructs to assess acceptability and we have used five of these constructs to guide our interpretation of the results of this study (Table 2).

**TABLE 2:** Interpreting the experiences of attending FAME using the Theoretical Framework of acceptability

Construct	Theme	Explanation
Affective attitude	Expectations and preparation for FAME	Anticipated affective attitude: expectations of and how an individual feels about taking part ahead of FAME
	The value of social support; FAME as a learning opportunity	Experienced affective attitude: how an individual feels about the programme, after participating in FAME
Burden	The value of social support; FAME as a learning opportunity	Experienced burden: the amount of effort that was required to participate for example, the exercise component or the difficulty managing challenging group dynamics
Intervention coherence	Expectation and preparation for FAME; FAME as a learning opportunity	The extent to which an individual understands the FAME programme, its contents and how it works that is, its aims and the contents of the programme
Perceived effectiveness	Expectation and preparation for FAME; The value of social support; FAME as a learning opportunity	Anticipated and experienced extent to which FAME is likely to/has achieved its aims
Self‐efficacy	Expectation and preparation for FAME; The value of social support; FAME as a learning opportunity	The patient or the HCP confidence of their capacity to perform the behaviours required by FAME

Abbreviation: FAME, Fibromyalgia active management and exercise programme.

Affective attitude (how an individual feels about the intervention before or after taking part) was broadly positive for most HCP participants, who valued the aims, format and content of FAME and perceived that most patient participants benefitted from attending the programme. However, patient participants expressed mixed views. Crucially, some participants reported that the programme was not what they expected, and this influenced their experience and acceptability of the programme. The coherence (the extent an individual understands the intervention, its contents and how it works) and perceived effectiveness (anticipated and experienced extent to which the intervention is likely to/has achieved its aims) of FAME were linked.

Expectations have been found to be predictors of treatment responses for many conditions including heart disease (Barefoot et al., 2011), surgery (Auer et al., 2016) and low back pain (Hayden et al., 2019). People with more positive treatment expectations are more likely to benefit from treatment (Laferton et al., 2017). Participants who understood and anticipated the aims and content of FAME reported that the programme was broadly acceptable and effective. This view was shared by all HCP participants but not the patient participants. Over ambitious expectations have been found to be correlated with reduced satisfaction with treatment outcomes (Mannion et al., 2009) and health outcomes (Oettingen, 2012). Therefore, the expectations about treatment response need to be in line with the aims of the FAME programme rather than ideal expectations or fantasies (Laferton et al., 2017). In addition, expectations regarding structural and process‐related aspects of the treatment are also likely to influence treatment coherence and acceptability (Laferton et al., 2017). This was reflected by some patient participants who expressed the desire for individual treatment approaches, were challenging FAME sessions, inconsistent staffing and lack of programme workbook. Thus, streamlining administrative processes, ensuring attendees understand the aims and content of FAME and are active participants in the treatment decision making process may optimise the programme acceptability and treatment outcomes.

Patient participants reported that support from peers and family and friends influenced their attendance at FAME (i.e. perceived emotional and/or instrumental (practical) support) and that the programme influenced their confidence to tackle challenging situations and treatments (e.g. physical activity). It is proposed that social support influences self‐efficacy (the confidence of the HCP or patient of their capacity to perform necessary behaviours and activities) and this could affect the acceptability of FAME. Informational support from the HCPs was also highlighted by some patient participants as important, although inconsistent staffing compromised the therapeutic relationship at times.

This study confirms the importance of social support for people with long term conditions (Bearne et al., 2020). People with fibromyalgia experience low levels of social support and significantly more loneliness than people with other Rheumatic diseases (Kool & Geenen, 2012). Lack of validation of their condition or illness legitimacy has been proposed to be interconnected to their physical symptoms with detrimental effects upon their illness and disability (Ghavidel‐Parsa & Bidari, 2021). In contrast, high quality social support networks have been associated with improved psychological wellbeing and increased levels of self‐efficacy for function and symptom management (Franks et al., 2004). Thus, FAME helped provide validation and support and helps explains why the role of social support was considered a crucial beneficial aspect of the programme. Our findings are concordant with a qualitative study on peer support in women with fibromyalgia (Sallinen et al., 2011) who found that peer‐support provided an impetus for reconstruction of identity, acceptance and coping with fibromyalgia.

Whilst education (informational support and learning) can reassure patients, legitimise symptoms and support self‐management (Rooks et al., 2007), learning from peers was seen as more credible by some of our patient participants than learning from HCP. This is corroborated by a qualitative study including 11 fibromyalgia patients found that sharing information with peers improved the speed at which patients could access useful information about the condition by explaining things in an understandable and relatable way (van Uden‐Kraan et al., 2008) potentially explaining why learning from peers was such a valuable part of FAME to participants.

Learning about mindfulness, but not the educational or exercise components, was universally valued by patient participants. Mindfulness has been demonstrated to be acceptable and beneficial in terms of pain, awareness, sleep, attention and wellbeing, in people with persistent pain and the evidence for fibromyalgia is promising (Haugmark et al., 2019). However, some participants commented on the high burden (the amount of effort that was required to participate) of some programme components (e.g. the perceived effort to exercise) and other patient participants reported they were already very active and this diminished the acceptability of the programme.

The education components were viewed by some patient participants as too generalised and a more individualised approach required. Individualised treatment approaches have been found to be valued in other persistent pain conditions (Lin et al., 2020; Wilson et al., 2016) and could be considered as an adjunct to FAME to optimise perceived effectiveness and acceptability. A recent systematic review found the best multi‐disciplinary treatments for fibromyalgia contained education, physical activity, pharmacology, cognitive behavioural therapy, and exercise programs with stretches and aerobic exercise (Llàdser et al., 2021). Whilst this reinforces the theme *expectations and preparation for FAME*, refinement of FAME may be necessary to make the delivery of components feasible and more acceptable.

### 6.1 Implications for practice

The FAME programme is a new multi‐model programme for people with fibromyalgia. With some refinement, it provides a feasible and acceptable group‐based intervention that potentially improves self‐management and health outcomes of people with fibromyalgia. Possible refinements to the programme include preparing participants for FAME to ensure their expectations align with the programmes aims and including an introduction session with active ‘opt‐in’ element; including an expert patient to facilitate learning opportunities; offering complementary individualised treatment components; setting and emphasising clear ground rules for expected behaviour within the group; supplementary HCP training on facilitating groups; ensuring continuity of staffing throughout the programme; and resources, such as an workbook, to facilitate understanding and engagement with FAME. In addition, a programme review group, with patients with a range of disease duration, is recommended. It was beyond the scope of this qualitative service evaluation to analyse costings of FAME. Future service evaluations should consider this and explore the duration of the 12‐week programme.

## 7 STRENGTHS AND LIMITATIONS

This evaluation had a number of strengths: a robust qualitative approach was undertaken with generation of rich data from patients and HCP; independent researchers conducted the interviews and HCP focus group, the themes were developed by a team and were interpreted using a theoretical model of acceptability all increasing validity of our findings.

There are some limitations. While all FAME attendees were invited to participate; the views of patients who did not attend all FAME sessions or those who declined to attend FAME were not captured. The patient participants interviewed had all had fibromyalgia for a long time and we were unable to include people with more recently diagnosed Fibromyalgia. Neither FAME nor the topic guide were coproduced with patients. Had FAME been designed in partnership with people with fibromyalgia, acceptability may have been enhanced from the outset. Furthermore, developing the topic guide with patients may have led to different avenues of enquiry and ensured we asked about subjects pertinent to them.

## 8 CONCLUSION

FAME is a new multi‐modal management programme for people with fibromyalgia. It was broadly acceptable for HCPs and some patient participants. Expectations and preparation for FAME, social support and the opportunity to learn about fibromyalgia and new treatment approaches all influenced the acceptability of FAME. Adaptation, including working collaboratively with stakeholders, is required to optimise acceptability for people with fibromyalgia.

Robustly evaluating acceptability of FAME from the perspective of people who receive and deliver it is an essential initial component before the scalability and sustainability is evaluated.

## CONFLICT OF INTEREST

The author declares that there is no conflict of interest that could be perceived as prejudicing the impartiality of the research reported.

## AUTHOR CONTRIBUTION

Suzanne McIlroy and Lindsay Bearne conceptualised the study, Suzanne McIlroy, Bethany Vaughan and Heather Crowe undertook the focus groups and interviews. All authors were involved in analysing the data and developing the themes. The manuscript was drafted by Suzanne McIlroy. All authors contributed to and reviewed the final manuscript.

## ETHICS STATEMENT

Ethical approval was not required but the protocol registered with the King’s College Hospital service evaluation and audit committee.

## Supporting information

Supplementary Material

## Data Availability

The datasets generated during and/or analysed during the current study are not publicly available. If required please contact the corresponding author.
