# Supplementary material for: The experiences and acceptability of a novel multimodal programme for the management of fibromyalgia: A qualitative service evaluation
Source: Musculoskeletal Care. 2022 Jul 15;20(3):686–96. doi: 10.1002/msc.1672 (PMC9545101; doi:10.1002/msc.1672)
Supplement: Supplementary file 1 — Supplementary Material [file MSC-20-686-s001.docx]

**Supplementary files**

1. Content of FAME sessions
2. Referral Criteria for Fibromyalgia Active Management and Exercise (FAME)
3. Topic guides

| **File 1: Content of FAME programme** | |
| --- | --- |
| **Week:** | **Session title:** |
| 1 | Introduction to FAME^1^  Education to pain and fibromyalgia^1, 2^  Introducing mindfulness^1, 2, 3^ |
| 2 | Identifying and setting goals^1, 2^  Body scan^1^  Understanding your medication discussion (offer of 1:1 follow-up)^2^ |
| 3 | The benefits of physical activity^1^  Exercise circuit programme^1^  Progressive muscle relaxation^1^ |
| 4 | Pacing^1, 2^  Chair based exercise^1^  Mindfulness^1, 2, 3^ |
| 5 | Motivational interviewing^1, 2, 3^  Reviewing goals^1, 2, 3^  Exercise circuit^1^  Visualisation exercise^1^ |
| 6 | What is mindfulness?^3^  Role of mood and emotions with pain^3^  Mindfulness exercise^3^  Pilates or chair based exercises^1^ (participant preference) |
| 7 | Sleep hygiene^1, 2^  Introduction to Tai chi/ mindful movement^1^ |
| 8 | Independent exercise in the community:  Suggestions:   - Walking group - Group hydrotherapy session |
| 9 | Pain coping strategies and modalities^1, 2^  Setbacks and preparing for self-management^1, 2^  Role of the pain team^2^  Tai chi/Mindful movement or exercise circuit^1^ (participant preference)  Visualisation exercise^1^ |
| 10 | Diet and nutrition^4^  Putting together an individual pain management toolkit^1, 2^  Exercise component^1^ – patient choice  Mindful breathing^1^ |
| 11 | Developing social support/ networks & relationships^3^   - *You may bring one family member or friend*   Exercise component^1^ – participant preference |
| 12 | Reviewing goals and progress^1, 2, 3^  Exercise component^1^ – patient choice |
| Session led by: 1 physiotherapist; 2 pain nurse; 3 psychologist; 4 dietician | |

| File 2: Referral Criteria for Fibromyalgia Active Management and Exercise (FAME) |
| --- |
| - Diagnosis of Fibromyalgia or widespread Pain and Fatigue - ≥ 6 months duration of symptoms - ≥ 18 years old - Conversational English - Cognitive Capacity - Committed to 12-week outpatient programme - Completed diagnostic pathway for pain - Able to mobilise safely in a group setting - Medically stable |

| **File 3: Example topic guides** |
| --- |
| **Patient Topic Guide** |
| What were you expecting from the FAME programme? |
| How did you find taking part in the FAME programme?   - Tell me about your experience of the programme |
| What were your views on the topics and content of the programme? |
| How did you find the format of the programme? |
| Tell me about what’s happened since you last attended with regards to your condition? |
| Do you have any recommendations for us to consider? |
| **Health care practitioner Topic Guide** |
| Please could you tell me about your contribution to the programme. |
| What were your expectations of the programme? |
| What did you learn from the experience? |
| How did you find taking part in delivering the FAME programme? |
| How did you feel your role fitted with the programme? |
| Please can you tell me your thoughts on the selection of the patients for this programme? |
| Has taking part in the FAME programme changed your view on referring patients to this class? |
| What did you think of the format of the FAME programme? |
| How did you find working within an MDT whilst delivering this programme? |
| Did you feel well prepared to take this programme? |
| Were you familiar with the outcome measures used? |
| Did you feel they were appropriate and relevant? |
| Are there any you would prefer to use? |
| Do you have any suggestions or recommendations for us to consider? |
